# Quality of End-of-Life Cancer Care in Canada: A 12-Year Retrospective Analysis of Three Provinces’ Administrative Health Care Data Evaluating Changes over Time

**DOI:** 10.3390/curroncol28060394

**Published:** 2021-11-12

**Authors:** Amanda Farah Khan, Hsien Seow, Rinku Sutradhar, Stuart Peacock, Kelvin Kar-Wing Chan, Fred Burge, Kim McGrail, Adam Raymakers, Beverley Lawson, Lisa Barbera

**Affiliations:** 1 Tom Baker Cancer Centre, University of Calgary, Calgary, AB T2N 4N2, Canada; amanda.khan@ucalgary.ca; 2 Institute for Clinical Evaluative Sciences, McMaster University, Hamilton, ON L8S 4K1, Canada; seowh@mcmaster.ca; 3 Institute for Clinical Evaluative Sciences, University of Toronto, Toronto, ON M5T 3M6, Canada; rinku.sutradhar@ices.on.ca; 4 Faculty of Health Sciences, Simon Fraser University, Burnaby, BC V5A 1S6, Canada; stuart_peacock@sfu.ca; 5 Odette Cancer Centre, Sunnybrook Health Sciences Centre, Toronto, ON M4N 3M5, Canada; kelvin.chan@sunnybrook.ca; 6 Department of Family Medicine, Dalhousie University, Halifax, NS B3H 4R2, Canada; fred.burge@dal.ca (F.B.); Bev.Lawson@Dal.Ca (B.L.); 7 Centre for Health Services and Policy Research, University of British Columbia, Vancover, BC V6T 1Z3, Canada; kim.mcgrail@ubc.ca; 8 British Columbia Cancer Agency, Vancover, BC V5Z 1L3, Canada; araymakers@bccrc.ca

**Keywords:** palliative care, quality indicators, health services research, cancer care, end of life

## Abstract

This retrospective cohort study of cancer decedents during 2004–2015 examined end-of-life cancer care quality indicators (QIs) in the provinces of British Columbia (BC), Ontario, and Nova Scotia (NS). These included: emergency department use, in-patient hospitalization, intensive care unit admissions, physician house calls, home care visits, and death experienced in hospital. Ontario saw the greatest 12-year decrease in in-hospital deaths from 52.8% to 41.1%. Hospitalization rates within 30 days of death decreased in Ontario, increased in NS, and remained the same in BC. Ontario’s usage of aggressive end-of-life measures changed very little, while BC increased their utilization rates. Supportive care use increased in both NS and Ontario. Those who were male or living in a lower income/smaller community (in Ontario) were associated with a decreased likelihood of receiving supportive care. Despite the shift in focus to providing hospice and home care services, approximately 50% of oncology patients are still dying in hospital and 11.7% of patients overall are subject to aggressive care measures that may be out of line with their desire for comfort care. Supportive care use is increasing, but providers must ensure that Canadians are connected to palliative services, as its utilization improves a wide variety of outcomes.

## 1. Introduction

Quality indicators are measurable items of health care performance that can be used to identify effective health care interventions or pinpoint areas of concern. For those with cancer, patient-defined outcomes that are important at the end of life include being physically independent for as long as possible, having adequate symptom control, spending time with friends and family, and dying at home or outside of hospital [1]. Patients who experience poor end of life (EOL) care are those who suffer from pain, are subjected to treatments that are overly burdensome, have their emotional/spiritual needs go unmet, or die in a setting outside of their home. In addition, their caregivers are often less able to move on after their death [1,2,3].

Administrative health care data has been employed to understand quality indicators of EOL care in numerous countries, such as the United States, Japan, Sweden, Belgium, and the United Kingdom [4,5,6,7]. However, the specific quality indicators used vary, which limits the ability to compare quality across services or settings [8]. In light of this, a systematic review published by Henson et al. evaluated 260 unique quality indicators of cancer care and found that of these, only 80 quality indicators received adequate testing to be appropriate for performance metrics. Furthermore, only 15 of these 80 quality indicators were highlighted as being scientifically sound and applicable across multiple care settings and domains of care [8].

Many jurisdictions in Canada have made commitments to improve end-of-life care, with a focus on more patient- and family-centered decisions and more support for palliative care [9]. As a result, we hypothesized that hospital deaths and other indicators of aggressive care would decrease and that indicators of supportive care would increase over the past decade. However, whether these commitments have in fact translated into tangible, measurable improvements in quality indicators needs to be elucidated. For instance, a 2015 Quality of Death Index report by the Economist Intelligence Unit showed that Canada slipped from 9th to 11th out of 80 countries based on availability, affordability, and quality of palliative care [10]. This paper analyzes end-of-life care for people dying of cancer across three Canadian provinces, over a 12-year time period, to determine whether there are changes in palliative care practices over time in several of the validated QIs outlined by Henson et al.

## 2. Materials and Methods

### 2.1. Population and Study Design

This cohort study retrospectively studied patients with the known cause of death attributable to cancer, between 1 April 2004 and 31 March 2015, across three different Canadian provinces: British Columbia (BC), Ontario, and Nova Scotia. The total population of these provinces together comprises 55% of the Canadian population as a whole. Exclusion factors included patients who were less than 18 years of age at time of death and those whose health card number was deemed invalid. In Ontario, only 624 patients were excluded due to these factors. Excluded patients for Nova Scotia and British Columbia were not obtained due to the lack of access to this information in the data request process.

### 2.2. Data Sources

All deaths attributed to a cancer diagnosis were identified from each province’s cancer registry. This included the BC Cancer Agency, the Ontario Cancer Registry, and the Nova Scotia Cancer Registry [11]. All registries utilized capture at least 90% of all new cancer cases for their respective province. Patient health card numbers were used to link cases to administrative health databases so that patient health services at EOL could be obtained. Data could not be analyzed in aggregate across provinces as this was not allowed due to each individual provinces’ privacy and confidentiality laws. Instead, data was analyzed by province and provincial rates compared to each other.

The databases used included the Discharge Abstract Database (which is overseen by the Canadian Institute for Health Information), provincial physician billing claims, and homecare databases [11,12,13].

Demographic information for Nova Scotia alone was obtained through that province’s cancer registry; for Ontario and BC, it was recorded from public health insurance records. The Statistics Canada 2006 national census was used to obtain neighborhood income and community size. A modified Deyo-Charlson Comorbidity Index (DCCI) score was computed using ICD10 codes from 24 to 12 months prior to death to analyze predicted mortality [14]. The modified DCCI for this analysis excluded points allotted to cancer. Data acquisition for each reported indicator is the same as previous manuscripts in order to facilitate comparison [2].

### 2.3. Health Service Quality Indicators

QIs that are widely used and identified as important to quality care at EOL by Henson et al. were considered for this study [8]. Those that were suitable for measurement using administrative data were included, specifically patients that had ≥1 new hospitalizations in the last month of life, ≥1 emergency department visits in the last 2 weeks or 30 days of life, an intensive care unit admission in the last month of life, and/or ≥1 physician house calls in the last 2 weeks of life. In addition, receipt of palliative homecare, nursing, and supportive services were examined among those who were eligible to receive such services during the last 6 months of life. Denominators for each indicator only included those at risk of the outcome being measured. For example, patients admitted for the entire final 30 days of life could not experience an ED visit and were excluded from the denominator of that indicator.

Aggregate measures of aggressive or supportive care were created by combining selected indicators, as done in prior research [2]. “Aggressive care” was defined as any one of or a combination of the following: two or more emergency department visits in the last 30 days before death, at least two new hospitalizations within 30 days of death, or an ICU admission within the last 30 days of life. “Supportive care” was defined as having at least one of or a combination of the following: a physician house call in the last 2 weeks before death, or a palliative nursing or personal support visit at home in the last 6 months before death. Supportive care data for BC was not included, as data for this measure was incomplete.

### 2.4. Statistical Analysis

Population characteristics at death were analyzed using descriptive statistics for each province. Overall crude and standardized rates for each indicator were calculated to allow interprovincial comparisons for the years 2004–2015. Crude rates were calculated as the proportion of patients who met that indicator’s definition. Standardized rates were calculated using the direct method and compared to 2014/2015 for the standard populations [2]. Two multivariable logistic regression models were created to understand what factors were associated with a patient receiving either aggressive or supportive care. Year of death was the main exposure, and other co-variates in the adjusted models were controlled. These co-variates included age, sex, score on the modified Deyo-Charlson comorbidity index, cancer type, neighborhood income quintile, community size, and health service region. Age was factored in as a continuous variable while the other variables were factored as categorical. Odds ratios (ORs) have been reported with 95% confidence intervals (CIs). Cochran–Armitage trend test *p*-values (two-sided) were calculated for all trend data. For cancer type, lung cancer was chosen as the reference cancer group because it was the most common cancer type and, therefore, the largest cancer subgroup.

Statistical analyses were performed using SAS (SAS Institute, Cary, NC, USA), R (The R Foundation, Vienna, Austria), and Microsoft Excel (Redmond, WA, USA). NumPy was utilized for data visualization [15]. The Hamilton Integrated Research Ethics Board (#3039) approved this study for Ontario data with the provision that all research must be conducted in accordance with the policies of the Institute for Clinical Evaluative Sciences. The Nova Scotia Health research ethics board, the Nova Scotia Department of Health and Wellness, and Health Data Nova Scotia approved the collection of data in that province, while the University of British Columbia (BC) Cancer Agency Research Ethics Board provided authorization in British Columbia.

## 3. Results

### 3.1. Demographics

In total, 376,108 patients who died from their cancer were included in this study and subsequently analyzed (Table A1). The mean age at death was 71.9 ± 12.7 years, and 46.9% of our study population were women. Overall age, sex, income, and cancer types were similar across the three different provinces. However, of note is that British Columbia had a slightly lower percentage of patients with a score of at least 1 on the Deyo-Charlson comorbidity index (34% compared to 39.4% in Ontario and 41.1% in Nova Scotia), and Nova Scotians live in smaller-sized communities.

### 3.2. Quality Indicators

Crude and standardized quality indicator rates (for all study years overall) are shown in the Appendix A in Table A2, by province. Overall, 50.1% of patients died in hospital; British Columbia had the lowest standardized rate of such deaths at 47.7%, whereas Nova Scotia had the highest rate at 66.5%. Patients with a new admission to hospital within 30 days of death ranged across provinces, from 49.5% in British Columbia to 57.6% in both Ontario and Nova Scotia. Rates of admission to the ICU were lowest in Nova Scotia at 4.0%, compared to 8.1% in Ontario. Comparing emergency department (ED) visit data within the last 30 days of death, British Columbia had the lowest use at 38.6%, whereas Ontario had the highest at 46.7%. Overall, 11.7% of all patients received aggressive care, with British Columbia having the lowest rate at 8.3%, with the highest in Ontario at 13.2%. Nova Scotia had the highest rate of supportive care usage between the two provinces with complete data at 55.3% (Ontario was 49.8%). Comparing Nova Scotia and Ontario, 54.7% of Nova Scotians received some form of palliative care at home vs. 46.7% of Ontarians, while 12.4% of Nova Scotians were visited by a physician within 2 weeks of death vs. 22.5% of Ontarians. British Columbia was excluded from the supportive care analysis due to incomplete data.

### 3.3. Data Trends over Time

**Figure 1:**
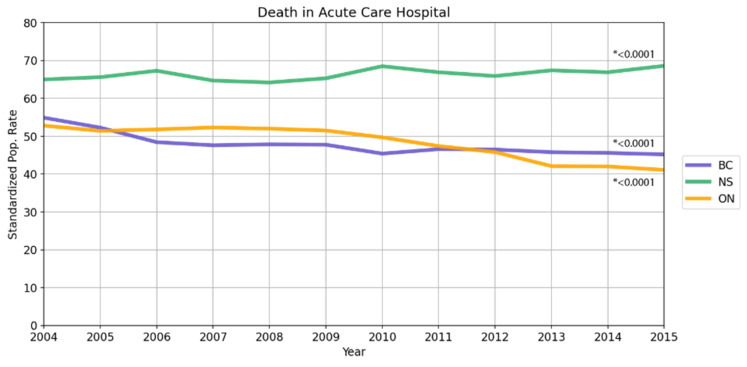

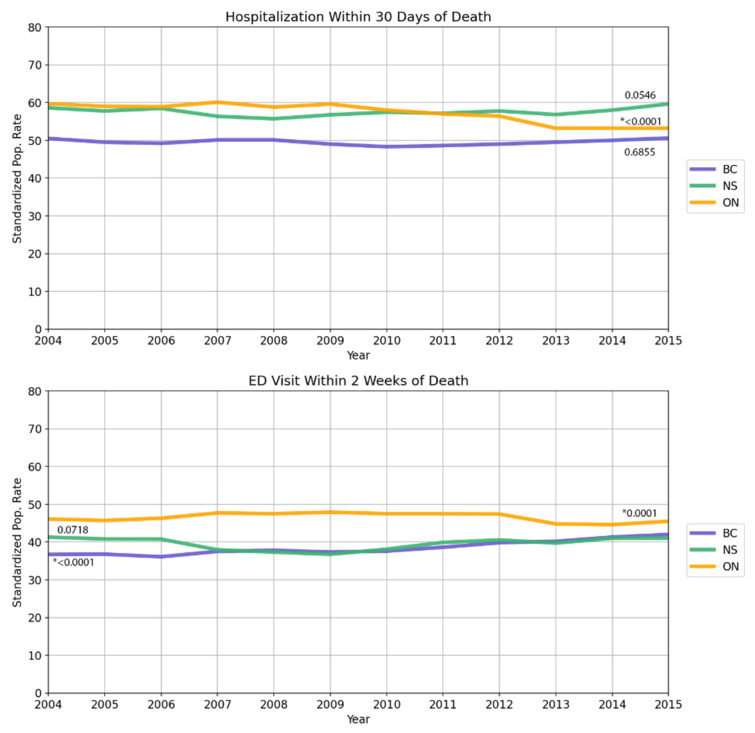
Death in acute care hospital (**top**), new hospitalizations within 30 days of death (**middle**), and one or more ED visits within 2 weeks of death (**bottom**) data over time from 2004 to 2015. In terms of death experienced in an acute care hospital, Ontario saw the greatest decrease, from 52.8% in 2004 to 41.1% in 2015. Ontario experienced the largest amount of change in hospitalizations within 30 days of death, decreasing from 59.7% in 2004 to 53.2% in 2015. Emergency room visits within 2 weeks of death stayed the same for Ontario and Nova Scotia, while British Columbia experienced an increase from 2004 to 2015. *p*-values reported are from the Cochran–Armitage trend test (two-sided); * denotes values that are statistically significant.

Data from 2004 to 2015 were analyzed for yearly trends over time. In terms of death experienced in an acute care hospital, Ontario experienced the greatest 12-year time span decline from 52.8% in 2004 to 41.1% in 2015 (*p* < 0.0001). British Columbia also experienced a decline from 54.9% to 45.2% in the same time span (Figure 1, top, *p* < 0.0001), while Nova Scotia experienced an increase in that time frame from 65% to 68.6% (*p* < 0.0001). In regard to hospitalization rates within 30 days of death, Ontario experienced the largest change, decreasing from 59.7% in 2004 to 53.2% in 2015 (*p* < 0.0001). Both Nova Scotia and BC rates were relatively stable during this time span (*p* > 0.05). Emergency room visits within 2 weeks of death were stable from 2004 to 2015 for Ontario (from 46.1% to 45.5%, *p* = 0.0001) and Nova Scotia (41.3% to 41.0%, *p* = 0.0718). British Columbia experienced an increase in ED visits from 36.7% to 42.0% (*p* < 0.0001).

By province, Ontario and BC largely remained consistent in their use of aggressive care measures (from 13.59% in 2004 to 13.43% in 2015, *p* = 0.2152, and from 7.8% in 2004 to 8.73% in 2015, *p* < 0.0001 respectively), while Nova Scotia decreased their use from 11.9% to 9.4% in the same time span (*p* < 0.0001). For the remaining years of analysis, Ontario saw an increase in supportive care usage from 44.0% in 2005 to 57.6% in 2015 (*p* < 0.0001). Nova Scotia also experienced an increase, from 54.3% in 2006 to 57.6% in 2015 (*p* < 0.0001).

### 3.4. Multivariable Logistic Regression Models

**Figure 2:**
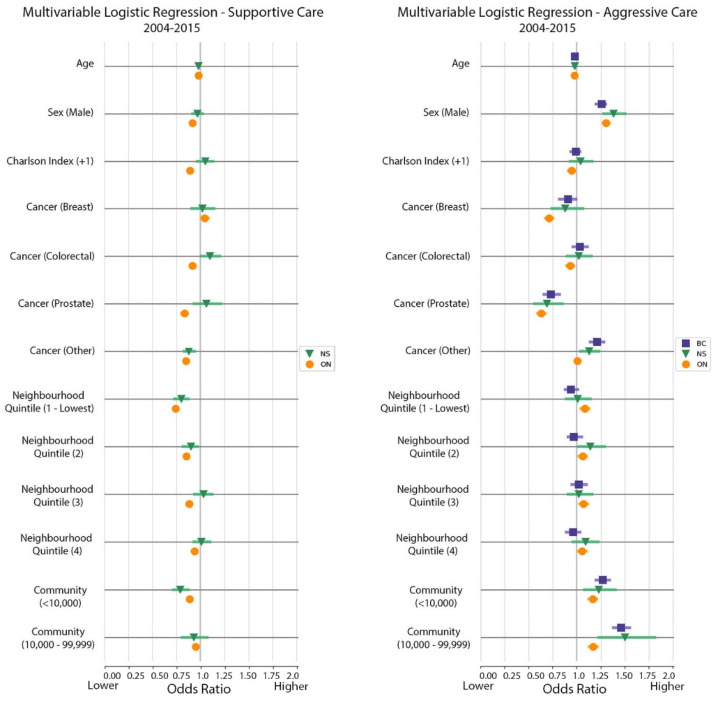
Multivariable logistic regression for supportive vs. aggressive care between 2004 and 2015. Reference odds ratio values are 1 for sex (female), Charlson Index (0 or missing), cancer type (lung), neighborhood income quintile (5 or highest), and community size (≥100,000).

The regression analyses showed that younger age, male sex, and residence in smaller-sized communities were all associated with an increased likelihood of receiving aggressive care (Table A3 and Figure 2)—an observation that was consistent for all provinces. In Ontario, living in a low-income neighborhood was also associated with receipt of aggressive care, whereas those with breast (OR of 0.71, 95% CI 0.82–0.99), colorectal (OR of 0.93, 95% CI 0.89–0.97), or prostate cancer (OR of 0.63, 95% 0.59-0.67) were less likely to receive aggressive care when compared to patients with lung cancer.

**Figure 3:**
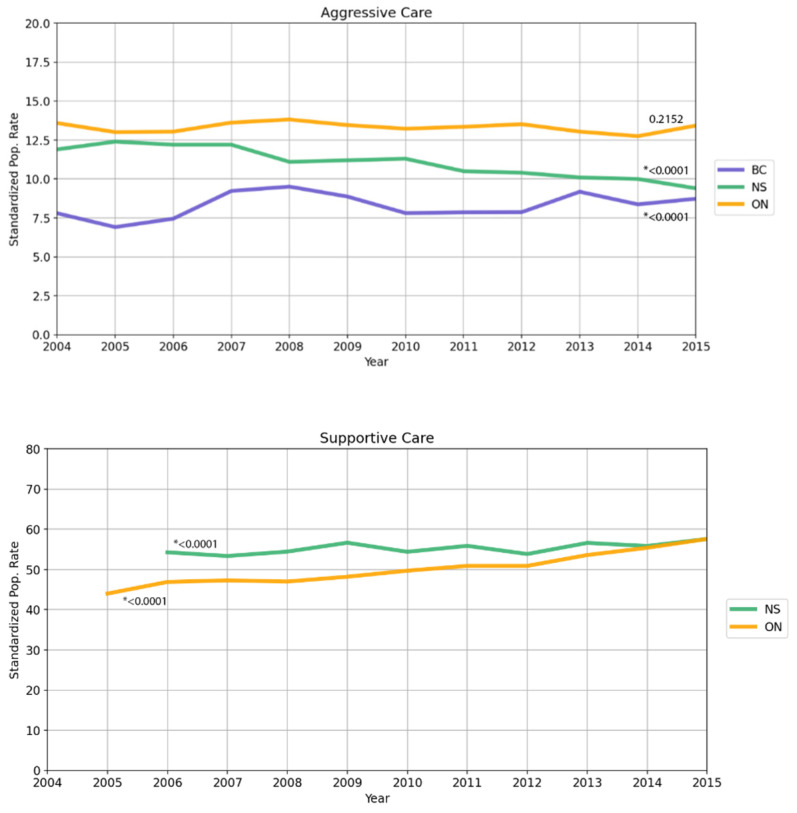
Aggressive care (top) and supportive care (bottom) data over time from 2004 to 2015. In Ontario, there was little overall change in the overall rate of receiving aggressive care (from 13.6% in 2004 to 13.4% in 2015). Aggressive care utilization dropped in this time in Nova Scotia from 11.9% in 2004 to 9.4% in 2015. British Columbia’s aggressive care utilization slightly increased from 7.8% in 2004 to 8.7% in 2015. Supportive care use rose in Ontario from 44% in 2004 to 57.6% in 2015. Nova Scotia’s utilization slightly increased from 54.3% in 2006 to 57.6% in 2015. *p*-values reported are from the Cochran–Armitage trend test (two-sided); * denotes values that are statistically significant. Ontario does not have reported data for 2004 and Nova Scotia does not have data for both 2004 and 2005.

In terms of analysis by year (Table A3 and Figure 3), understanding the trends in the utilization of aggressive care is not as straight forward as the supportive care model. Nova Scotians over time utilized aggressive care less, which correlated to decreased odds ratios from 2004 to 2015. Ontario experienced little change in the standardized rates of aggressive care usage, which is consistent with the relatively steady odds ratios in the multivariable study. BC data shows the most variability in standardized rate over time, which is reflected in the variation in odds ratios in the regression model for this province.

Factors associated with an increased likelihood of receiving supportive care amongst the three provinces were younger age and female sex (Table A4 and Figure 2). Living in a larger community was also associated with a higher likelihood of receiving supportive care. In Ontario, those with a score of at least 1 on the Deyo-Charlson Comorbidity Index were less likely to receive supportive care. Of note, compared with people in the highest-income neighborhoods, people living in the lowest-income neighborhoods had a 0.74–0.80 (CI of 1.04 to 1.13) likelihood of receiving supportive care. Also in Ontario, those diagnosed with breast cancer had a higher likelihood of receiving supportive care when compared to patients with lung cancer. In Nova Scotia those with colorectal cancer had an increased likelihood of receiving supportive care than those with lung cancer. In terms of analysis by year (Table A4), access to supportive care improved over time for both Ontario and Nova Scotia, as odds ratios trend upwards toward 2015 as the reference point, which parallels the overall improvement in standardized rates plotted in Figure 2.

## 4. Discussion

We present a 12-year analysis of commonly defined quality indicators in EOL care across three Canadian provinces using administrative health care data to create identically defined cohorts. This paper represents the most comprehensive and current analysis of end-of-life QIs for cancer patients in Canada. It provides longitudinal data including multiple care settings, which have been prior research gaps. Examining quality specifically among cancer patients is important because cancer care has a long history of incorporating palliation, and cancer has a more defined end-of-life trajectory compared to other terminal chronic illnesses, such as COPD, which may mean greater access to supportive care at EOL [15]. Patients with end-stage cancer also often have high rates of hospitalization, but the use of aggressive end-of-life care is often discordant with the desires of the general population [16]. Yet, across many countries, most patient deaths still occur in an institutional setting instead of at home [17,18]. While acute care hospitals serve a role in managing the needs of oncology patients at the end of life, quality end of life care can be provided in other settings, including at home or in hospice by family physicians, home care nurses, and personal care workers [19].

In our analysis, we found inconsistent evidence of improvement. Fewer Canadians are dying in hospital over time, particularly in BC and Ontario. Hospitalization rates within 30 days of death in Ontario have also improved over time, possibly due to a funding strategy that enhanced access to end-of-life home care services, but to define a conclusive cause of improvement is a complex and nuanced discussion [20]. During the same period, Nova Scotia’s hospitalization rate worsened, while BC largely remained the same. Reducing unnecessary hospitalizations is of particular interest to publicly funded health care systems, as the overall cost of cancer care is rising, with a high proportion of dollars being spent on end-of-life care [16]. In NS and Ontario, indicators related to supportive care also seem to be improving. Again, these may reflect investments made into these sectors in combination with increased awareness and emphasis on the role supportive care services can play, but further research into the causality is needed.

Regardless of any observed improvements over time, all indicators remain worse than previously published Canadian benchmarks [21]. For example, the benchmark rate (based on regional top performers) for death in hospital was 38%, yet in our analysis, all three provinces were consistently worse than these benchmarks. This was also true for other indicators. The benchmark rate for ED visits in the last 30 days was 34%, ICU admissions in the last 30 days was 2%, house calls in the last 2 weeks was 34%, and home care in the last 6 months was 63%. This indicates that while some gains have been made, opportunities for further improvement remain. In contrast to studies from other countries, Canadian in-hospital death rates remain higher than those in the United States, but are similar to those in Taiwan and Singapore, and lower than those in Norway [22,23,24].

Our multivariable logistic regression models showed that patients who were male, younger, and living in poorer neighborhoods or smaller communities across the three provinces are less likely to receive supportive care services. This is an important finding, as research shows that Canadians who come from low-income, rural, or new immigrant backgrounds have poorer access to high-quality palliative care [25,26]. Our data remains consistent with data reported by other Canadian studies in regards to hospitalization rates and socioeconomic and location discrepancies in aggressive vs. supportive care [22,27].

Limitations of this study include the fact that not all data were available in each province. For example, chemotherapy information is not available from Nova Scotia and is available from very different sources in Ontario (claims) and BC (pharmacy data); thus, chemotherapy use was not included in our study. Similarly, there were differences in data sources for other measures such as ED that compromised comparability. For this reason, inpatient hospitalization data were used to identify patients admitted to hospital via the ED, which was consistent but did not count ED visits that did not lead to hospitalization. Lastly, the indicators themselves have limitations and may not capture important aspects of care. For example, it did not reflect patient quality-of-life, caregiver burden, or preferences. There is also inconsistent or unavailable data for other QIs that Henson et al. outlined as important, such as physical aspects of care (including those who received tube or intravenous feeding in the month before death) or the number of patients who received opioids in the month before death [8].

## 5. Conclusions

This work highlights 12 years of quality EOL data across three different Canadian provinces with identically identified cohorts and commonly defined QIs. We have found strong evidence that despite the shift in focus to providing earlier EOL care to oncology patients with supportive care measures, approximately half of oncology patients are still dying in hospital, although it is improving. Furthermore, 11.7% of patients are subject to aggressive care measures that may be out of line with their desire for comfort care in a non-hospital setting. Our study highlights that over the 12-year time span studied, little change occurred in the use of aggressive care services and ED visits within the last 2 weeks of life. However, supportive care use is on the rise, with Ontarians increasing their use in our study time frame. Access to robust palliative care services is important for symptom management, particularly at end of life. By not connecting patients to palliative and supportive care services at end of life, they may miss out on evidence-based interventions and services that are known to improve a wide variety of outcomes.

## Data Availability

All data used in the preparation of this paper is governed by each respective province and is not publicly available due to privacy and regulations governing the use of these data.

## Appendix A

**Table A1:** Socio-demographic data by province, 2004–2015.

Characteristic	BC		ON		NS		Overall	
	*n*	%	*n*	%	*n*	%	*n*	%
Study Population	104,106		242,556		29,446		376,108	
Age group								
18–49 Years	4984	4.8	12,782	5.3	1401	4.8	19,167	5.1
50–79 Years	65,234	62.7	154,257	63.6	18,877	64.1	238,368	63.4
80+ Years	33,878	32.5	75,517	31.1	9168	31.1	118,563	31.5
Sex								
Male	55,381	53.2	128,772	53.1	15,559	52.8	199,712	53.1
Income quintile								
1 (lowest)	23,094	23.1	51,526	21.3	6300	21.7	80,920	21.9
2	20,459	20.5	51,569	21.4	6170	21.3	78,198	21.1
3	19,380	19.4	47,424	19.6	5864	20.2	72,668	19.6
4	18,418	18.4	46,496	19.3	5595	19.3	70,509	19.0
5 (highest)	18,532	18.6	44,382	18.4	5070	17.5	67,984	18.4
Community size								
>1,500,000	44,829	43.8	80,513	33.2	0	0	125,342	33.5
500,000–1,499,999	0	0	31,359	12.9	0	0	45,255	12.1
100,000–499,999	18,050	17.6	66,341	27.4	13,896	47.8	87,860	23.5
10,000–99,999	23,126	22.6	27,855	11.5	3469	11.9	62,681	16.8
<10,000	16,363	16.0	36,306	15.0	11,700	40.3	53,050	14.2
Cancer type								
Brain	2856	2.8	6679	2.8	713	2.4	10,248	2.7
Breast	7245	7.0	18,908	7.8	1949	6.6	28,102	7.5
Colorectal	12,781	12.4	30,397	12.5	3964	13.5	47,142	12.6
Gynecological	4483	4.3	11,138	4.6	1176	4.0	16,797	4.5
Head and Neck	2394	2.3	6650	2.7	625	2.1	9669	2.6
Hematology	9616	9.3	25,098	10.3	2190	7.4	36,904	9.8
Lung	26,447	25.7	66,784	27.5	8337	28.3	101,568	27.1
Other	13,164	12.8	21,879	9.0	4264	14.5	39,307	10.5
Other Gastrointestinal	11,864	11.5	26,497	10.9	2998	10.2	41,359	11.0
Other Genitourinary	5952	5.8	12,778	5.3	1705	5.8	20,435	5.4
Prostate	6304	6.1	15,748	6.5	1525	5.2	23,577	6.3
Score on the Deyo-Charlson comorbidity index (from 24 months to 12 months before death)								
0	55,008	52.8	116,225	47.9	14,492	49.2	185,725	49.4
1+	35,401	34.0	95,510	39.4	12,098	41.1	143,009	38.0
Missing	13,697	13.2	30,821	12.7	2859	9.7	47,377	12.6

**Table A2:** Quality indicator rates by province, 2004–2015.

Indicator	BC		ON		NS		Overall	
	*n*	%	*n*	%	*n*	%	*n*	%
		Std		Std		Std		Crude
c	104,106		242,556		29,446		376,108	
Death in acute care hospital or bed (overall)								
	49,687	47.7	119,037	48.9	19,627	66.5	188,351	50.1
New Hospitalization within 30 days of death								
≥1 New admission	51,701	49.5	129,091	57.6	16,857	57.6	197,649	55.4
With new intensive care unit admission	3136	3.0	16,685	8.1	1181	4.0	21,002	5.9
All eligible patients (excluding those in hospital for the last 30 days of life)	104,014		223,325		29,109		356,448	
Emergency department visit within 30 days of death								
Source: CIHI Discharge Abstract Database	40,200	38.5	104,428	46.7	11,571	39.7	156,199	43.8
All eligible patients (excluding those in hospital for the last 30 days of life)	104,014		223,325		29,109		356,448	
Home visit within 6 months of death								
By a registered nurse			156,118	64.9	12,804	51.6		
By a personal support worker			98,992	41.2	5047	20.5		
Having a palliative nursing or personal support worker home care visit within 6 months of death			98,534	46.0	13,514	54.9		
All eligible patients (excluding those in hospital for the last 30 days of life)			240,500	-	24,630	-		
Physician house call within 2 weeks of death								
Patients who received a house call			53,521	27.3	3586	12.4		
All eligible patients (excluding those in hospital for the last 14 days of life)			196,061	-	28,964	-		
Aggressive care								
At least one care factor listed below:	8776	8.3	29,802	13.2	3283	11.1	41,861	11.7
Having at least 2 ED visits within 30 days of death	3872		10,201		1000		15,073	4.2
Having at least 2 new hospitalizations within 30 days of death	6913		15,023		2235		24,171	6.8
Being in the ICU within 30 days of death	7122		18,293		1181		26,596	7.5
All eligible patients (excluding those in hospital for the last 30 days of life)	104,014		223,325		29,109		356,448	-
Supportive care								
Utilizing at least one service below:			107,353	50.1	13,701	55.3		
Having a physician house call within 2 weeks of death			48,238	22.5	3586			
Having palliative nursing or personal support worker home care visit within 6 months of death			98,534	46.0	13,514	54.7		
All eligible patients (excluding those in hospital for the last 6 months of life)			214,389		24,630			

**Table A3:** Multivariable logistic regression model for aggressive care, 2004–2015.

Factor	BC			ON			NS (New Health Zone)		
	OR	95% CL		OR	95% CL		OR	95% CL	
		Lower	Upper		Lower	Upper		Lower	Upper
Age (years)	**0.98**	**0.97**	**0.98**	**0.98**	**0.98**	**0.98**	**0.98**	**0.97**	**0.98**
Male (reference: Female)	**1.26**	**1.20**	**1.30**	**1.31**	**1.27**	**1.34**	**1.38**	**1.28**	**1.5**
Charlson comorbidity index 1+ (reference: 0 or missing)	**0.99**	**0.94**	**1.03**	**0.95**	**0.91**	**0.98**	1.04	0.93	1.16
Cancer type (reference: Lung)									
Breast	**0.91**	**0.82**	0.99	**0.71**	**0.68**	**0.75**	0.88	0.74	1.06
Colorectal	1.03	0.96	1.11	**0.93**	**0.89**	**0.97**	1.02	0.9	1.15
Prostate	**0.73**	**0.66**	**0.82**	**0.63**	**0.59**	**0.67**	**0.69**	**0.56**	**0.85**
Other	1.21	1.14	1.28	**1.01**	**0.98**	**1.04**	**1.13**	**1.04**	**1.23**
Neighborhood income quintile (reference: 5, highest)									
1 (lowest)	0.94	0.88	1.01	**1.09**	**1.04**	**1.13**	1.01	0.89	1.14
2	0.98	0.91	1.05	**1.06**	**1.02**	**1.10**	**1.14**	**1.02**	**1.29**
3	1.02	0.95	1.1	**1.07**	**1.03**	**1.11**	1.02	0.91	1.16
4	0.96	0.89	1.03	**1.05**	**1.013**	**1.10**	1.09	0.96	1.22
Community size (reference: ≥100,000)									
<10,000	**1.27**	**1.21**	**1.34**	**1.17**	**1.13**	**1.21**	**1.23**	**1.08**	**1.4**
10,000–99,999	**1.47**	**1.38**	**1.55**	**1.17**	**1.13**	**1.22**	**1.5**	**1.23**	**1.81**

Boldface type indicates significant values; OR—odds ratio; CL—confidence limits.

**Table A4:** Multivariable logistic regression model for supportive care, 2004–2015.

Factor	ON			NS (New Health Zone)		
	OR	95% CL		OR	95% CL	
		Lower	Upper		Lower	Upper
Age (years)	**0.98**	**0.98**	**0.98**	**0.98**	**0.98**	**0.98**
Male (reference: Female)	**0.92**	**0.90**	**0.93**	**0.97**	**0.92**	**1.02**
Charlson comorbidity index 1+ (reference: 0 or missing)	**0.89**	**0.87**	**0.91**	**1.05**	**0.97**	**1.13**
Cancer type						
Breast	**1.04**	**1.01**	**1.08**	1.02	0.91	1.14
Colorectal	**0.92**	**0.89**	**0.95**	**1.10**	**1.01**	**1.20**
Prostate	**0.84**	**0.80**	**0.87**	1.06	0.93	1.21
Other	**0.85**	**0.83**	**0.87**	**0.88**	**0.83**	**0.94**
Lung	1	-	-	1	-	-
Neighborhood income quintile (reference: 5, highest)						
1 (lowest)	**0.74**	**0.72**	**0.76**	**0.80**	**0.73**	**0.87**
2	**0.85**	**0.83**	**0.88**	**0.90**	**0.82**	**0.97**
3	**0.88**	**0.86**	**0.91**	1.03	0.94	1.12
4	**0.94**	**0.91**	**0.96**	1.01	0.93	1.10
Community size (reference: ≥100,000)						
<10,000	**0.89**	**0.87**	**0.91**	**0.79**	**0.72**	**0.87**
10,000–99,999	**0.95**	**0.93**	**0.98**	0.93	0.81	1.07

Boldface type indicates significant values, OR = odds ratio, CL = confidence limits.
